# Endogenous Opioid Release After Orgasm in Man: A Combined PET/Functional MRI Study

**DOI:** 10.2967/jnumed.123.265512

**Published:** 2023-08

**Authors:** Patrick Jern, Jinglu Chen, Jouni Tuisku, Tiina Saanijoki, Jussi Hirvonen, Lasse Lukkarinen, Sandra Manninen, Semi Helin, Vesa Putkinen, Lauri Nummenmaa

**Affiliations:** 1 Department of Psychology, Åbo Akademi University, Turku, Finland;; 2 Turku PET Centre, University of Turku, Turku, Finland;; 3 Turku University Hospital, University of Turku, Turku, Finland;; 4 Department of Radiology, University of Turku, Turku, Finland;; 5 Turku Institute for Advanced Study, University of Turku, Turku, Finland;; 6 Department of Psychology, University of Turku, Turku, Finland; and; 7 Turku BioImaging, University of Turku and Åbo Akademi University, Turku, Finland

**Keywords:** orgasm, manual penile stimulation, arousal, opioids, fMRI, PET

## Abstract

The endogenous μ-opioid receptor (MOR) system plays a key role in the mammalian reward circuit. Human and animal experiments suggest the involvement of MORs in human sexual pleasure, yet this hypothesis currently lacks in vivo support. **Methods:** We used PET with the radioligand [^11^C]carfentanil, which has high affinity for MORs, to quantify endogenous opioid release after orgasm in man. Participants were scanned once immediately after orgasm and once in a baseline state. Hemodynamic activity was measured with functional MRI during penile stimulation. **Results:** The PET data revealed significant opioid release in the hippocampus. Hemodynamic activity in the somatosensory and motor cortices and in the hippocampus and thalamus increased during penile stimulation, and thalamic activation was linearly dependent on self-reported sexual arousal. **Conclusion:** Our data show that endogenous opioidergic activation in the medial temporal lobe is centrally involved in sexual arousal, and this circuit may be implicated in orgasmic disorders.

## Visual Abstract

**Figure:**
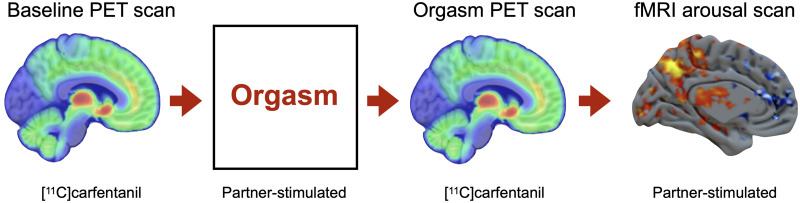

The endogenous opioid system plays a key role in the mammalian reward circuit (1), and the opioid receptor also regulates sexual desire and arousal (2). Opiates suppress sexual behaviors in both humans and animals (3), and μ-opioid receptor (MOR) agonists, in particular, decrease human sexual desire and pleasure acutely and chronically (4). The roles of opioid receptor agonists and antagonists in exciting and suppressing sexual behaviors is complex and varies between species and conditions. Opioid antagonists and agonists may also promote sexual behavior: naltrexone stimulates ejaculations and increases copulation rates in male rats (5). Opioid agonists may also induce copulation when injected to the medial preoptic area (6), whereas striatal administration does not, at least not consistently (7). A recent study also showed that MOR availability in cortical and subcortical areas correlated positively with sex drive in men (2). Animal studies also demonstrate postcoital endogenous opioid release: copulation releases endogenous opioid peptides in rats in the medial preoptic area of the hypothalamus (8). In humans, opioid agonists increase pleasure, and opioid abusers describe the sensations after opioid administration as euphoric and orgasmic (9). Accordingly, evidence suggests that the opioid receptor contributes to human sexual drive and pleasure, but in vivo evidence for endogenous opioid release after sexual behaviors is lacking. Here, because of the potential implications in orgasmic disorders, we tested the hypothesis that sexual arousal peaking in orgasm leads to endogenous opioid release in men.

## MATERIALS AND METHODS

**FIGURE 1.**
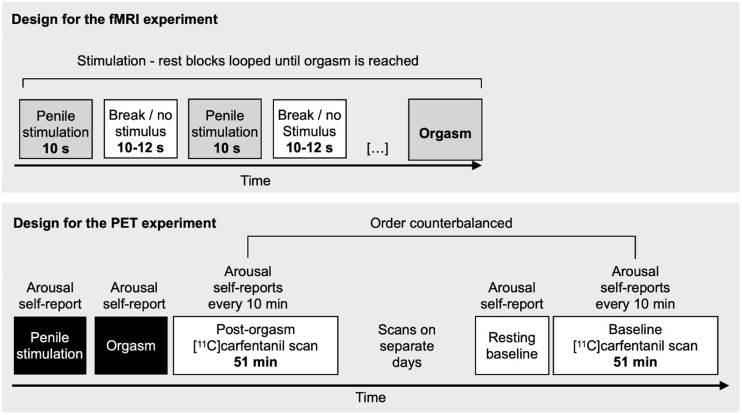
Depiction of study design. fMRI = functional MRI.

The participants were 6 heterosexual males (mean age, 35.4 y, range, 21.6–43.2 y). Participants gave informed, written consent and were compensated for their participation. The Hospital District of Southwest Finland’s ethics board approved the protocol. The study was conducted in accordance with the Declaration of Helsinki. The participants’ female partners served as confederates, providing tactile penile stimulation (scanning details are provided in the supplemental materials (available at http://jnm.snmjournals.org). The participants were scanned with PET at baseline and after receiving orgasm-leading penile stimulation. The scans were done on separate days, and their order was counterbalanced (Fig. 1, top row). The mean time between PET and MRI scans was 14.67 d (SD, 12.59 d). The couple was led to a private room 30 min before the orgasm scan; the partner was instructed to time the participant’s orgasm as close to the PET scan as possible. MOR availability was measured with the radioligand [^11^C]carfentanil (10), synthesized as described previously (11). Regional time–activity curves were obtained from 21 regions of interest, and regional nondisplaceable binding potential values were obtained for each scan using the simplified reference tissue model with the occipital cortex as a reference (Supplemental Table 1) (10–22). PET imaging was performed with a Discovery 690 PET/CT scanner. The tracer was administered as a single bolus via a catheter placed in each participant’s antecubital vein. PET emission data were acquired for 51 min after injection. Regional nondisplaceable binding potentials across the orgasm and baseline conditions were compared using paired-sample *t* tests without multiple-comparison correction across regions of interest.

The MRI data were acquired using a Phillips Ingenuity TF PET/MRI 3-T scanner. The participant was covered with a blanket, and the partner was sitting next to the MRI bed. The partner received auditory instructions to stimulate her partner in approximately 10-s blocks (Fig. 1). The participant used buttons to indicate his moment-to-moment sexual arousal (on a scale of 0–100). Stimulation blocks were interspersed with approximately 11-s rest blocks (±1-s jitter). Functional data were preprocessed with FMRIPREP (version 1.3.0) (15) and analyzed using a general linear model with t-contrast for manual penile stimulation versus rest as well as moment-to-moment parametric modulation of sexual arousal. All results were corrected using a false-discovery-rate *P* value of less than 0.05.

## RESULTS

**FIGURE 2.**
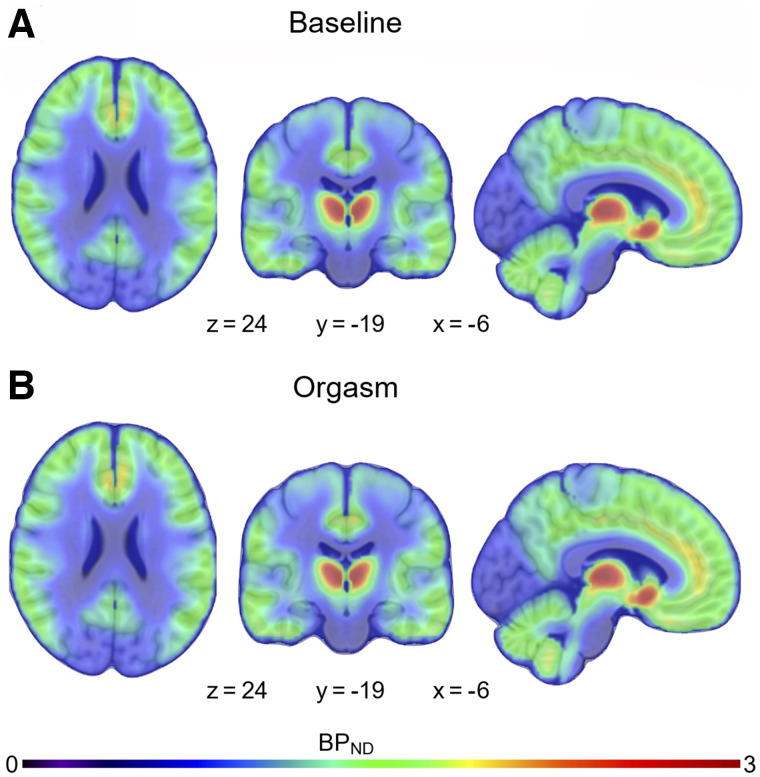
Mean MOR availability during baseline and orgasm scans.

**FIGURE 3.**
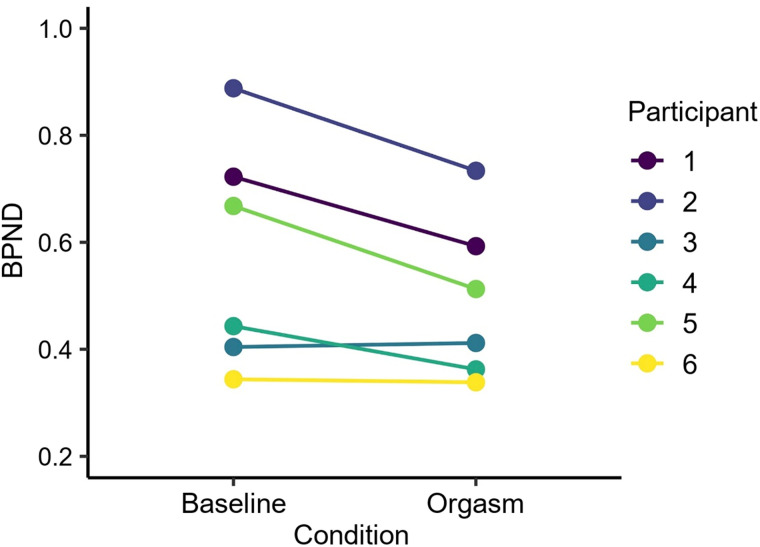
Mean participantwise nondisplaceable binding potential in hippocampus for baseline and orgasm scans. Difference is statistically significant at *P* < 0.05 (paired *t* test). BPND = nondisplaceable binding potential.

**FIGURE 4.**
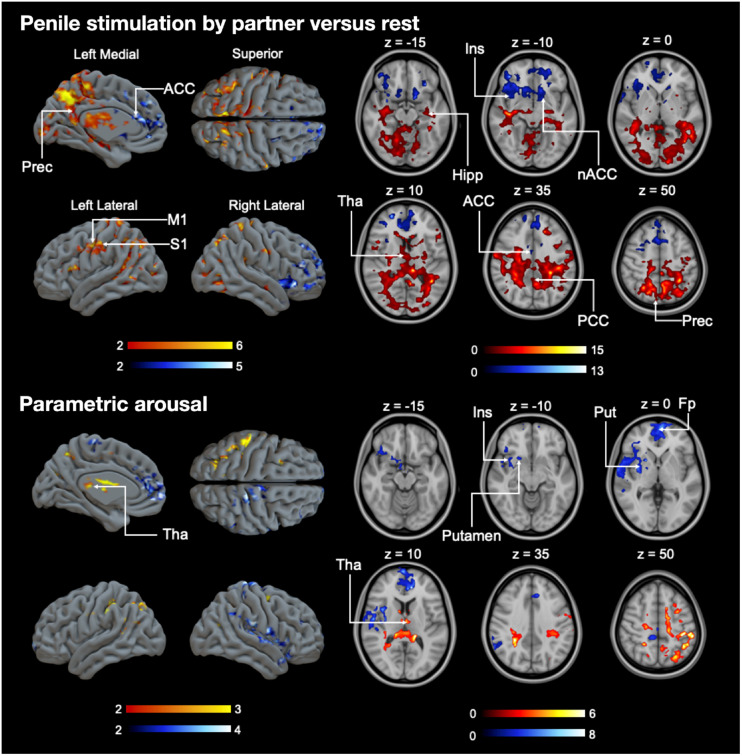
(Top) Brain regions showing amplified responses during manual penile stimulation minus rest. (Bottom) Brain regions whose activity increased linearly as function of sexual arousal during scans. Data are thresholded at *P* < 0.05, false-discovery-rate–corrected at cluster level. Color bar indicates *t* statistic range. ACC = anterior cingulate cortex; Hipp = hippocampus; Ins = insula; Fp = frontal pole; Put = putamen; M1 = primary motor cortex; nACC = nucleus accumbens; PCC = posterior cingulate cortex; Prec = precuneus; S1 = primary somatosensory cortex; Tha = thalamus.

The mean pleasure rating for the orgasm was 8.1 units, and the mean arousal rating was 6.33 units (scale, 1–10 units; Supplemental Fig. 1). Arousal after the orgasm was significantly higher than during any other time point (*t*s > 3.27, *P*s < 0.02 in paired-sample *t* test). Figure 2 shows mean MOR availability during baseline and orgasm scans. The region-of-interest analysis (Fig. 3) yielded a significant effect in the hippocampus (*t*(5) = 2.90, *P* = 0.03), indicating endogenous opioid release after orgasm (Δ*M* = 13%). No other significant effects were observed. Sexual arousal increased linearly during functional MRI (fMRI) scans (Supplemental Fig. 2). Hemodynamic responses to receiving penile stimulation minus rest were stronger in the hippocampus and thalamus and in the posterior parietal, primary motor, and somatosensory cortices. Deactivations were observed in the anterior cingulate cortex in the nucleus accumbens and insula (Fig. 4A). The parametric model for pleasure yielded positive effects in the thalamus and inferior parietal cortices. Negative associations were observed in the insula, putamen, and frontal pole (Fig. 4B).

## DISCUSSION

We provide an indication that in vivo endogenous opioid release may be raised in the male hippocampus after orgasm. Orgasm led to increased opioid release in the medial temporal lobe. Hemodynamic activity during penile stimulation increased in limbic regions and the somatosensory cortex, whereas responses in the thalamus reflected the moment-to-moment intensity of sexual arousal.

PET data revealed higher nondisplaceable binding potential in the hippocampus in the baseline versus orgasm scan, consistent with increased neurotransmitter release after an orgasm. These changes were paralleled by hemodynamic responses in the hippocampus during penile stimulation. The fMRI results also implied a central role of the thalamus in modulating sexual arousal. To our knowledge, these data yield the most detailed picture, to date, of the functional and molecular brain basis of sexual arousal and climax in man and support the general role of the MOR system in modulating the calmness–arousal axis (2*,*^23^).

Opioid release after sexual activity corroborates findings of increased opioid receptor activity after sexual behaviors in other mammals (8). This finding parallels human PET studies demonstrating endogenous opioid release after consumption of rewards ranging from feeding to sociability (24) and extends the role of the human MOR system to sexual pleasure. Data were also in line with recent PET data indicating MOR-dependent individual differences in male sex drive, corroborating the role of MORs in sexual motivation and pleasure (2). Because of the temporal resolution of PET, we cannot, however, state whether opioid release reflects pleasure evoked by sexual stimulation, orgasm, or refractory activity in the postorgasmic phase.

Perhaps surprisingly, orgasm-dependent MOR activation was observed only in the hippocampus. However, animal electrophysiologic studies have demonstrated hippocampal activity during orgasm (25). Furthermore, θ-hippocampal activity can be induced by injecting opiates into the brain stem (26). Indeed, the rat brain may enter a learning mode during the postorgasmic phase, analogous to the memory consolidation that is known to occur during sleep after learning (27). We did not observe MOR activity in the hypothalamus despite its role in sexual functioning in rodents (6). This may reflect insufficient statistical power, yet we observed no hypothalamic effects even at lower statistical thresholds. The hypothalamus is, however, a small structure whose accurate quantification using PET with limited spatial resolution is complicated.

Brain activity increases during sexual reward in humans and animals. fMRI revealed increased activation in the hippocampus and thalamus during penile stimulation versus rest, the former according with increased MOR activation observed in the PET study. The thalamus modulates arousal and awareness (28), and the male thalamus becomes activated during penile erection, acting as a relay station transmitting peripheral sexual sensations to the brain (29). We also observed deactivation in the anterior cingulate and activation in the posterior parietal, primary motor, and somatosensory cortices. These areas have been shown to activate during sexual arousal and orgasm in men (30).

Our study had some limitations. Evidence for opioid release was found only in 1 region (hippocampus) of 21 a priori regions of interest, and although the hippocampus contains MORs, the regional nondisplaceable binding potentials were moderately low. Because full-volume false-discovery-rate–corrected results were not observed, the finding should be considered preliminary until replicated. Only men were studied, and the sample size was limited by the complex multimodal imaging setup; however, significant effects were nonetheless observed. Brain responses during the orgasm phase in fMRI could not be analyzed because of head motion.

## CONCLUSION

We observed endogenous opioid release in the male brain after orgasm in 6 healthy volunteers. In a parallel fMRI experiment, we observed activation of—above all—the hippocampus. Altogether, these data show that endogenous opioidergic activation in the medial temporal lobe is centrally involved in sexual arousal, whereas modulation of sexual arousal in the thalamus and striatum may be supported by other neuromodulators.

## DISCLOSURE

The study was supported by the Sigrid Juselius Stiftelse and Academy of Finland (294897, 332225). No other potential conflict of interest relevant to this article was reported.

## Notes

KEY POINTS **QUESTION:** Does sexual arousal peaking in orgasm lead to endogenous opioid release in men? **PERTINENT FINDINGS:** In a combined PET/fMRI study involving 6 healthy participants, we found evidence of opioid release in the hippocampus (*P* < 0.05). **IMPLICATIONS FOR PATIENT CARE:** Endogenous opioidergic activation in the medial temporal lobe is centrally involved in sexual arousal, and this circuit may be implicated in orgasmic disorders.
